# Improving Autism Spectrum Condition Support Within an Early Intervention Psychosis Service

**DOI:** 10.1192/bjo.2024.434

**Published:** 2024-08-01

**Authors:** Karen Collier, Sashriya Singh

**Affiliations:** Coventry and Warwickshire Partnership Trust, Warwick, United Kingdom

## Abstract

**Aims:**

To improve the knowledge of the early intervention in psychosis team staff regarding support and referral options available for patients with suspected or confirmed autism spectrum conditions. We further aimed to develop additional knowledge within this team to help identify, assess and support people who may be neurodivergent with a primary focus on autism.

**Methods:**

Starting in September 2022 the team completed a survey to understand baseline knowledge. Plan Do Study Act (PDSA) quality improvement cycles were then used to develop resources and disseminate knowledge within the team to help target the identified staff development areas. Following implementation the team completed a further survey to reassess the changes and ongoing areas of focus and to guide ongoing skill development.

**Results:**

3 PDSA cycles were completed with interventions including collecting data on local services to share within the team, collating these resources and sourcing training sessions. This demonstrated an improvement in many areas at the point of the second survey at which stage the team showed a better understanding of how to access autism assessments, the role of various local autism specific teams including admission avoidance urgent support processes and confidence in supporting people after diagnosis. Work continued following the second survey within the team with further training sessions and both medical and psychology colleagues upskilling to be able to complete autism assessments within our service in conjunction with the neurodevelopmental team. The early intervention team staff have been able to utilise the support of many of these services and often discuss these options now within team meetings.

**Conclusion:**

Initially support and knowledge gaps were identified within our team and work was done to collect and share information about local services and processes to best allow us to support those within our psychosis service who also have an autism spectrum condition and this has been successfully implemented.

This work has grown over the past 2–3 years since the initial quality improvement work was developed and team knowledge has since continued to grow. This has included multiple team members now also being able to complete autism assessments formally in conjunction with the neurodevelopmental service with some assessments now finalised and significant ongoing work to improve experiences of those with both autism and psychosis.

